# Comparison of the Influence of Oxygen Groups Introduced by Graphene Oxide on the Activity of Carbon Felt in Vanadium and Anthraquinone Flow Batteries

**DOI:** 10.1021/acsaem.3c03223

**Published:** 2024-03-18

**Authors:** Antonio J. Molina-Serrano, José M. Luque-Centeno, David Sebastián, Luis F. Arenas, Thomas Turek, Irene Vela, Francisco Carrasco-Marín, María J. Lázaro, Cinthia Alegre

**Affiliations:** † Instituto de Carboquímica, Consejo Superior de Investigaciones Científicas-CSIC. C/Miguel Luesma Castán, 4, 50018 Zaragoza, Spain; ‡ Institute of Chemical and Electrochemical Process Engineering, Clausthal University of Technology, Leibnizstraße 17, 38678 Clausthal-Zellerfeld, Germany; § Research Center for Energy Storage Technologies, Clausthal University of Technology. Am Stollen 19 A, 38640 Goslar, Germany; ∥ Facultad de Ciencias, Universidad de Granada. Avd. de Fuente Nueva, s/n, 18071 Granada, Spain

**Keywords:** 2,7-AQDS, electrocatalysis, energy storage, hexacyanoferrate, modified felt, organic flow battery, redox flow battery

## Abstract

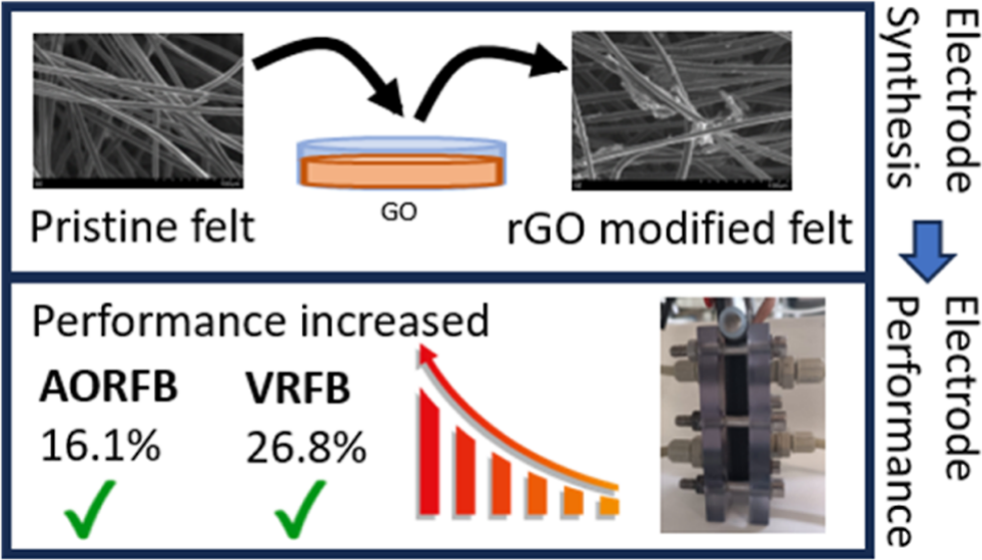

An increasing number of studies focus on organic flow batteries (OFBs) as possible substitutes for the vanadium flow battery (VFB), featuring anthraquinone derivatives, such as anthraquinone-2,7-disulfonic acid (2,7-AQDS). VFBs have been postulated as a promising energy storage technology. However, the fluctuating cost of vanadium minerals and risky supply chains have hampered their implementation, while OFBs could be prepared from renewable raw materials. A critical component of flow batteries is the electrode material, which can determine the power density and energy efficiency. Yet, and in contrast to VFBs, studies on electrodes tailored for OFBs are scarce. Hence, in this work, we propose the modification of commercial carbon felts with reduced graphene oxide (rGO) and poly(ethylene glycol) for the 2,7-AQDS redox couple and to preliminarily assess its effects on the efficiency of a 2,7-AQDS/ferrocyanide flow battery. Results are compared to those of a VFB to evaluate if the benefits of the modification are transferable to OFBs. The modification of carbon felts with surface oxygen groups introduced by the presence of rGO enhanced both its hydrophilicity and surface area, favoring the catalytic activity toward VFB and OFB reactions. The results are promising, given the improved behavior of the modified electrodes. Parallels are established between the electrodes of both FB technologies.

## 1 Introduction

The need for energy storage has increased as a result of escalating energy demand, atmospheric pollution, and climate change caused by carbon dioxide emissions. Stationary energy storage can help to satisfy this need and integrate renewable energy sources, such as wind, solar, or tidal power, into the electricity grid.^1^ However, the random and intermittent nature of renewable energy sources can cause complications and additional costs due to voltage and frequency fluctuations. Large- and medium-scale energy storage could solve these problems, allowing for efficiency, safety, fast-response time, and reasonable cost.^2^ Electrochemical energy storage provides numerous benefits, such as reliability, durability, manufacturability, and independence from geographical location.^3^

Flow batteries (FBs) are one of the most promising electrochemical technologies for large-scale energy storage. FBs can offer hour-length storage and flexible, fast-response operation both in load leveling and frequency regulation modes.^4^ FBs store energy in aqueous solutions of redox couples, making them safer by resisting thermal runaway and fire, offering a modular design that favors scalability as well as decoupled power and capacity.^5^ In particular, vanadium flow batteries (VFBs) have achieved commercial status and proven long lifetimes due to the use of vanadium species in both positive and negative electrolytes, which mitigates cross-contamination through the membrane.^6,7^ On the other hand, the implementation of VFBs has been limited by the elevated and fluctuating costs of raw minerals, not to mention the risky supply chains for this critical resource.^8^

As a result, much interest has been recently devoted to the development of FBs based wholly or partially on redox-active organic molecules (OFBs).^9,10^ It is presently considered that organic molecules could be produced from abundant, noncritical precursors, alleviating concerns about supply bottlenecks, mining impact, resource location, and shifting prices. Most research has focused on the development of stable viologen and anthraquinone derivatives for long-lifetime negative electrolytes.^11^ This is followed by the investigation of NO-radicals, thiazine, and quinone compounds for the positive electrolytes, although given their poor stability, activity has shifted toward metal complexes, such as ferrocyanide.^12^ One of the main challenges in OFBs is to emulate or surpass the electrochemical performance, energy efficiency, and service lifetime of VFBs. This challenge may be overcome not only by addressing electrolyte chemistry but also by adequately choosing cell components such as electrode materials and ion exchange membranes.^13^

Carbon-felt electrodes are one of the critical components of VFBs and OFBs based on fully soluble redox species. This porous material is used due to its low cost, good mechanical properties, high chemical stability, and large surface area.^14^ These characteristics contribute to the overall performance of the battery, allowing operation at a relatively high current density and reducing kinetic and mass transfer overpotentials.^15,16^ However, carbon felts are known to show aging effects (from hours to years, depending on the conditions), moderate electrical conductivity, and modest electrocatalytic activity, leading to limited energy efficiency in FBs.^17^ The modification of carbon felts with electrocatalysts is one of the main strategies to improve their characteristics as electrodes and overcome their limitations.^18,19^ These modifications can be carried out following different methods, for example, the insertion of metals such as Ir, Cu, and Pt^20,21^ or metal oxides such as Mn_3_O_4_ and WO_3_.^22,23^ Yet, metal-based modifications can be unstable in strongly acidic/basic and oxidizing/reducing electrolytes and can usually be unfeasible for cost-effective electrodes.

On the other hand, the insertion of carbonaceous materials to carbon felts in FBs may prove advantageous over metal-based modifications.^24^ Some carbon nanostructures like nanofibers or graphene have been investigated for VFBs due to their chemical stability, large specific surface area, and high electrical conductivity.^25^ High-surface area carbonaceous materials with enhanced electrocatalytic activity are generally less expensive to produce, namely, carbon nanofibers^25^ and carbon nanotubes.^26^ One of the main positive effects is the extension of the electrode/electrolyte interface by the increase in the hydrophilicity and the roughness derived from the incorporation of carbon nanomaterials on the felt surface.^27,28^ Moreover, these nonprecious materials can be doped or functionalized with heteroatoms such as oxygen or nitrogen^26,29,30^ or by introducing graphene oxide.^31,32^ Several studies have reported an enhanced FB performance due to the catalytic effect of the oxygen functional groups on the surface of carbon materials, which is generally accompanied by an increase in both the surface area and electrical conductivity of the electrode.^33,34^ However, achieving the application of carbon-modified electrodes without any performance fading is still a major challenge for long-term operation in practical FB systems.

The effect of these modifications on VFBs carbon felt electrodes has already been investigated,^18^ showing that they afford good results at low current densities, in particular for the positive electrode.^35^ The reactions of V^2+^/V^3+^ and VO^2+^/VO_2_^+^ redox couples and their mechanisms have been studied also at unmodified felt,^36,37^ showing that phenolic groups provide active sites that catalyze both the VO^2+^/VO_2_^+^ and V^2+^/V^3+^ redox reactions. In addition, it has been proposed that the charge and discharge processes at the positive electrode imply the transfer of an oxygen atom, which is likely to be the limiting reaction in the overall mechanism.^38^ This drawback can be solved by the surface addition of different oxygen functional groups.^16,39^ Indeed, improvement mechanisms depend specifically on the nature of the many electrocatalyst-modified felts proposed for VFBs.^18^

In strong contrast, the effect of modified carbon felts on the performance of OFBs has received little attention. For instance, Ni(OH)_2_-modified carbon felt has been proposed as an oxygen evolution catalyst at the positive electrode of the 3,3′-(9,10-anthraquinone-diyl)bis(3-methylbutanoic acid) (DpivOHAQ)/ferrocyanide FB for rebalancing purposes (without involving the organic redox couple).^40^ Another example is the treatment of carbon felt with nickel, iron, and cobalt chlorides for the 1,8-dihydroxyanthraquinone (DHAQ)/ferrocyanide system (vide infra).^41^ The scarcity of research on this topic may be understood on the basis of the numerous molecular analogues and the limited synthesis scale achieved for most of them.^42^ However, 2,7-AQDS is one of the few readily available organics used in prospective FBs, being found as a redox additive in the Stretford process for the desulfurization of natural gas.^43^ This molecule is thus applied in the 2,7-AQDS/ferrocyanide FB, which has received considerable interest.^44−46^ Still, only two studies have investigated the effect of carbon felt and its thermal modification on this chemistry. For instance, the Stokes radius of 2,7-AQDS has been compared to that of VOSO_4_ and K_4_[Fe(CN)_6_], showing that the organic molecule had the highest value among these species, resulting in slow transport to the fibers.^47^ By extrapolation, another work using the isomer 2,6-AQDS has noted that thermal activation of carbon felt delayed capacity loss by providing catalyst sites to recover intermediate degradation species by oxidizing them back into 2,6-AQDS.^48^

The objective of the present work is to investigate the modification of commercial carbon felts with reduced graphene oxide (rGO) and polyethylene glycol (PEG) on the 2,7-AQDS redox couple for the first time and to preliminarily assess its effects on the efficiency of the 2,7-AQDS/ferrocyanide FB. Results are compared to those of a VFB in order to evaluate if the benefits of the felt modification observed with this system are transferable to OFBs. The oxidizing power of PEG produces a carbon-felt surface rich in oxygen groups, increasing its catalytic activity. Plus, the presence of rGO confers enhanced catalytic activity toward the redox reactions. After their physical characterization, these modified electrode materials are evaluated first by cyclic voltammetry and then in flow batteries, aiming to reveal the improvement pathways for OFBs.

## 2 Experimental Section

### 2.1 Modification of Carbon Felt with Polyethylene Glycol and Reduced Graphene Oxide

**Figure 1:**
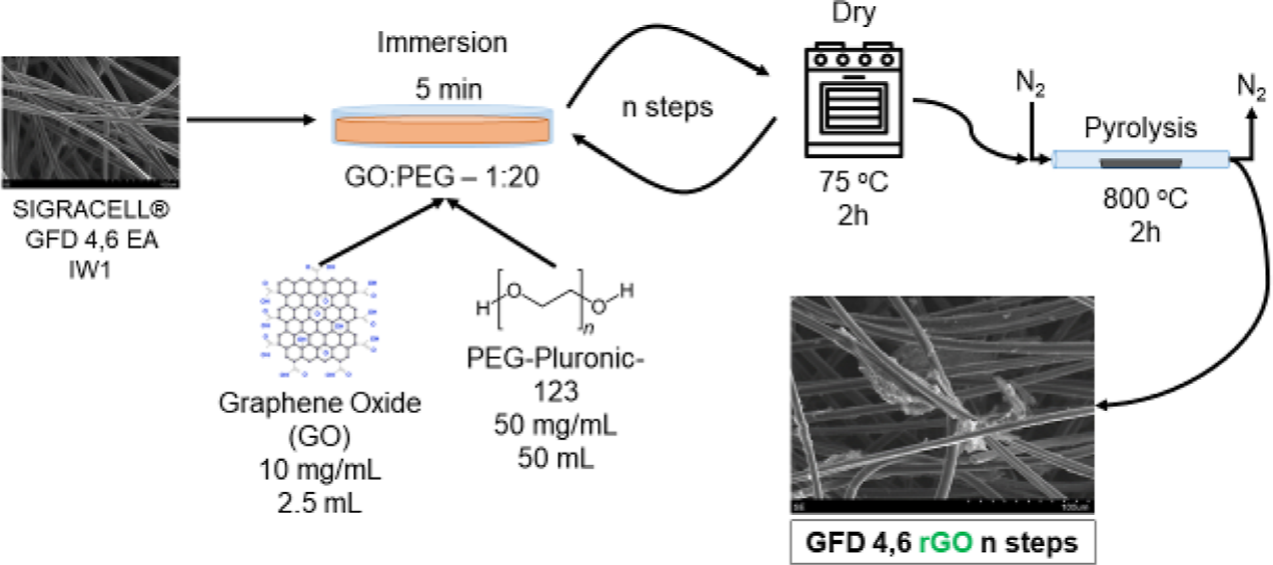
Scheme representing the modification process of the carbon felt by GO-PEG impregnation cycles.

A carbon felt based on carbonized and activated polyacrylonitrile fibers was selected for modification and sourced from SGL Carbon (Sigracell GFD 4.6 EA IWI, 4.6 mm thick). As shown in Figure 1, different pieces of the carbon felt were modified following a method based on impregnation with reduced rGO and PEG, followed by drying steps.^31^ Briefly, a PEG solution, Pluronic P-123 (Sigma-Aldrich), was first prepared with a concentration of 67.5 mg mL^–1^ in deionized water/ethanol (1:1 v/v). In a different beaker, a 10 mg mL^–1^ aqueous suspension of graphene oxide (GO) was prepared by sonication for 30 min in an Elmasonic S 60H bath (Elma Schmidbauer). GO was obtained by oxidation and exfoliation of graphite powder (C >99,8%, Sigma-Aldrich) following the Tour method.^49^ Afterward, 2.5 mL of the GO suspension was added to a Petri dish with 50 mL of the PEG solution. A piece of 6 cm × 6 cm (approximate mass of 1.11 g) of carbon felt was subsequently immersed in the GO-PEG solution of the Petri dish, carefully ensuring the full wetting of the material. After 5 min of immersion, the felt was removed and dried in an oven at 75 °C for 2 h under air. Once dried, the immersion and drying process were repeated 2, 5, 7, 10, and 12 times. Once these impregnation steps were completed, the resulting pieces of carbon felt were subjected to controlled pyrolysis for an additional 2 h at 800 °C in a nitrogen atmosphere.

### 2.2 Physicochemical Characterization

The chemical composition of the modified felts was determined by elemental analysis using a Flash 1112 analyzer (Thermo) and atomic emission spectrometry with inductively coupled plasma (ICP-AES) in an Xpectroblue-EOP-TI FMT26 instrument (Spectro). X-ray photoelectron spectroscopy (XPS) was carried out on an ESCA Plus spectrometer (Scientia Omicron) equipped with an Al (1486.7 eV) anode and a power of 225 W (15 mA, 15 kV). The deconvolution of O 1s and C 1s spectra was carried out with the CasaXPS software suite, considering 30% Gaussian and 70% Lorentzian peak shapes and a Shirley background. Morphology was evaluated by scanning electron microscopy (SEM) in an SEM 3400N microscope (Hitachi). Raman spectra of the modified carbon felt were obtained with an HR800 UV (HORIBA Scientific), using the green line argon laser (λ = 532 nm) as the excitation source and evaluating the relative intensities of D (ca. 1350 cm^–1^) and G (ca. 1590 cm^–1^) peaks. XRD measurements were carried out using a D8 ADVANCE diffractometer (Bruker) with Cu Kα radiation and a power of 1600 W.

### 2.3 Electrochemical Characterization

#### 2.3.1 Substances and Solutions

High-purity 9,10-anthraquinone-2,7-disulfonic acid disodium salt, 2,7-AQDS(Na_2_), was purchased from Dequenne R.D.I. The salt had a reported mass content of 96.1% 2,7-AQDS(Na_2_) and 2.8% of the isomer 9,10-anthraquinone-2,6-disulfonic acid disodium salt, 2,6-AQDS(Na_2_) according to high-performance liquid chromatography (HPLC). In order to remove any carbonate or bicarbonate residues,^46^ the salt was dissolved in batches of 20 g in 0.5 L of water, filtrated, and then dried in a stirred vessel placed in an oil bath at 80 °C until the mass of the sample acquired a stable value. Commercial vanadium electrolyte was obtained from HydraRedox Iberia S.L. and consisted of an aqueous solution of 0.4 M VO^2+^ and V^3+^ (1:1, initial state of charge (SOC): −50%), 2 M H_2_SO_4_, and 0.05 M H_3_PO_4_. All other substances were of analytical grade and purchased from Fluka, Carl Roth, or Merck and used without further purification. Deionized water with a conductivity of 18.2 MΩ cm was used in the preparation of all of the solutions.

#### 2.3.2 Cyclic Voltammetry

The activity of the modified felts toward the reactions of interest was investigated in a three-electrode polytetrafluoroethylene (PTFE) cell designed by Santamaría et al.^24,29^ The working electrode consisted of a circular piece of modified felt (projected surface area of 0.950 cm^2^) placed at the bottom of the cell, in contact with a graphite current collector. The reference electrode was Ag/AgCl (3 M KCl), and the counter electrode was a graphite bar. The felt was compressed to 50% of its original thickness with the aid of a threaded piece. A thorough wetting of the felt was achieved by repeated immersions in the electrolyte before its use in the PTFE cell. All experiments were carried out at room temperature (22 ± 1 °C) and were preceded by purging the electrolytes for 20 min with nitrogen (5.0 grade). The V^2+^/V^3+^ and VO^2+^/VO_2_^+^ redox reactions for the vanadium FB were investigated by using an aqueous solution containing 0.05 M VOSO_4_ and 1.0 M H_2_SO_4_ as the electrolyte and connecting the cell to an Autolab PGSTAT302N potentiostat (Metrohm). The 2,7-AQDS redox couple was studied by employing an electrolyte containing 0.05 M 2,7-AQDS in a 1.0 M (NH_3_)_2_SO_4_ solution, similar to the conditions reported by Fenini et al.^46^ In this case, the three-electrode cell was controlled with an SP-50 potentiostat (Biologic).

#### 2.3.3 Flow Battery

Experiments were carried out in membrane-divided flow cells in a two-electrode configuration. This rectangular channel flow cell design (projected surface area of 10 cm^2^, 2.5 cm × 4 cm) has been described elsewhere,^50^ although in this case, Viton gaskets with a thickness of 4.0 mm were used in order to accommodate a carbon felt electrode in each half-cell and compress them to 87% of their original thickness against 3 mm thick PPG86 graphite plates (Eisenhuth). The electrolytes were pumped to each half-cell at a rate of 50 mL min^–1^, which represents an average linear velocity through the felt of 1.0 cm s^–1^. All experiments were performed in a sealed flow system consisting of fluorinated ethylene-propylene tubing for the 30 mL glass vials or 100 mL HPLC flasks used as “tanks” and Viton tubing for the peristaltic pump. The room temperature during the experiments was 24 ± 1 °C. Charge and discharge were performed in a simple galvanostatic regime at increasing current densities, performing 10 cycles for each value after purging both electrolytes with nitrogen for 30 min. A nitrogen flow rate of 0.3 L min^–1^ was maintained in the tanks during the measurements.

For the VFB, 50 mL of vanadium electrolyte was employed for each half cell, charging the system from a SOC of −50 to 100% under a constant cell voltage of 1.6 V until a current of 50 mA was reached before the experiments. The flow cell had a Nafion 117 membrane with a dry thickness of 183 mm (Chemours), was fed by a D25 V2i peristaltic pump (Dinko), and was connected to a VSP-3e potentiostat coupled to a VMP3B-20 amplifier (Biologic). Cut-off cell voltage values were 1.8 V for charging and 0.4 V for discharging. OFB experiments were performed using 15 mL of a solution of 0.2 M 2,7-AQDS and 1.0 M (NH_3_)_2_SO_4_ as the limiting negative electrolyte and 30 mL of a solution of 0.2 M Na_4_[Fe(CN)_6_]·6H_2_O and 1.0 M (NH_3_)_2_SO_4_ as the nonlimiting positive electrolyte. The sealed flow system was protected from the ambient light. The flow cell for the OFB was fitted with a Nafion 211 membrane with a dry thickness of 51 μm (Chemours), a Sci-Q323 peristaltic pump (Watson Marlow), and a Reference 3000 potentiostat (Gamry). Cut-off cell voltage values were 1.1 and 0.3 V for charge and discharge, respectively.

## 3 Results and Discussion

### 3.1 Morphological and Chemical Characterization

**Figure 2:**
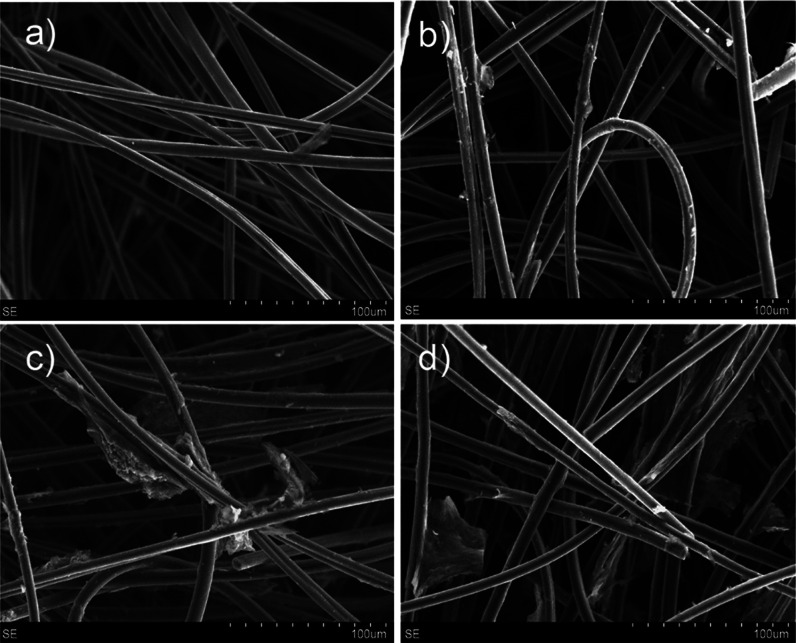
SEM images of rGO-PEG-modified graphite felt with a different number of impregnation steps: (a) pristine felt, (b) 5 steps, (c) 10 steps, and (d) 12 steps.

SEM images of the pristine and modified carbon felt are shown in Figure 2. The images reveal that the pristine carbon felt (Figure 2a) consists of tangled fibers of about 8 μm in diameter. The presence of rGO was detectable upon five impregnation steps with rGO-PEG. Figure 2b shows that the fibers of a felt modified by five impregnation steps are decorated with rGO, as indicated by the presence of amorphous particles. As expected, an increasing number of impregnation steps resulted in a larger coverage of rGO on the fibers (Figure 2c,d with 10 and 12 impregnation steps, respectively), which increased the overall roughness and surface area of the porous material.

The measurement of deposited rGO was determined by comparing the weight of modified samples of felt to pristine samples subjected to the same PEG wetting and controlled pyrolysis process. Table 1 shows the estimated amounts of rGO deposited after each impregnation step. These values indicate that after seven impregnation steps, the amount of rGO remains at a very similar loading, which implies that a higher number of impregnation steps does not result in an effective increase in the amount of deposited rGO.

**Table 1:** Amount of rGO Deposited on Each Material Calculated by Weight Compared to a Material Subjected to the Same Preparation Process but without the Use of GO in the Impregnation Steps

electrode material	mg rGO cm^–2^
pristine felt	0
2 steps	0.50
5 steps	1.49
7 steps	2.49
10 steps	2.98
12 steps	2.51

**Figure 3:**
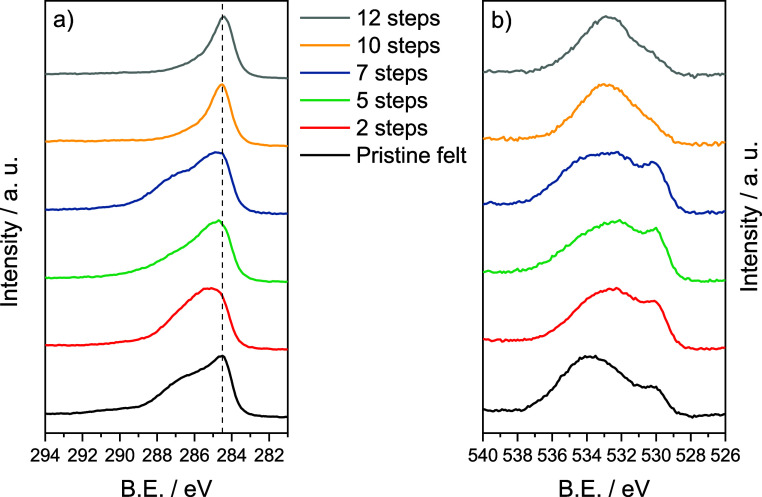
High-resolution XPS spectra of felts with an increasing number of impregnation steps. (a) C 1s and (b) O 1s.

The chemical composition of the modified felts was investigated by both X-ray photoelectron spectroscopy (XPS) and elemental analysis (EA). Figure 3a,b shows the comparison between the high-resolution C 1s and O 1s spectra, respectively, for the felts characterized by a different number of steps incorporating rGO-PEG.

In the C 1s high-resolution spectra shown in Figure 3a, the position of C=C sp^2^ carbon species is indicated with a dashed line at a binding energy of 284.5 eV. By comparing all the spectra, some differences in the evolution of the C species are made evident. These differences have been determined by deconvolution of each spectrum (Figure S1) and are reported in Table S1. At a low number of impregnation steps (2–7), there is a variety of surface C species with a great amount of oxygen groups, such as C–OH and C–O–C species appearing at 286.1 and 288.4 eV, respectively. C=O and COOH species found at 287.2 and 289.4 eV, respectively, are also encountered, although in a lower amount with respect to the former. Besides oxidized carbon species, the main peaks from C–C sp^2^ (284.5 eV) and C–C sp^3^ (285.3) are also found on the surface of the modified felts.^32,51^ On the other hand, from 10 steps of rGO/PEG deposition, the number of species decreases radically, presenting a main peak of C=C sp^2^ band and lower contribution of C–C sp^3^, C–OH and C–O–C species compared with the felts modified with less impregnation steps.

Figure 3b shows the high-resolution O 1s spectra for the pristine felt and the evolution of the modified felts with the increasing number of impregnation steps. Like in the C 1s high-resolution spectra, the shape of the spectrum changes with the amount of rGO incorporated that originates from different oxygen species. Oxygen species distribution was calculated from the deconvolution of the high-resolution O 1s spectra (see Supporting Information, Figure S2). In the pristine carbon felt, there are four main bands at 530.1, 532.0, 533.6, and 535.1 eV, which correspond to the presence of O–C–OH, O=C, OH–C, and adsorbed H_2_O species, respectively.^32,51^ The atomic percentage of each species is reported in Table S1, showing how the amount of O–C–OH and C=O species significantly increases from the pristine carbon felt to the felts with five impregnation steps confirming the incorporation of rGO sheets. From 10 impregnation steps, the main species found are O=C and OH–C, in accordance with the results obtained by Di Blasi et al., who suggested that the formation of these species is a consequence of the reduction of C–O–C groups in the presence of C–OH in the same basal plane, allowing the formation of phenolic groups.^51^

**Figure 4:**
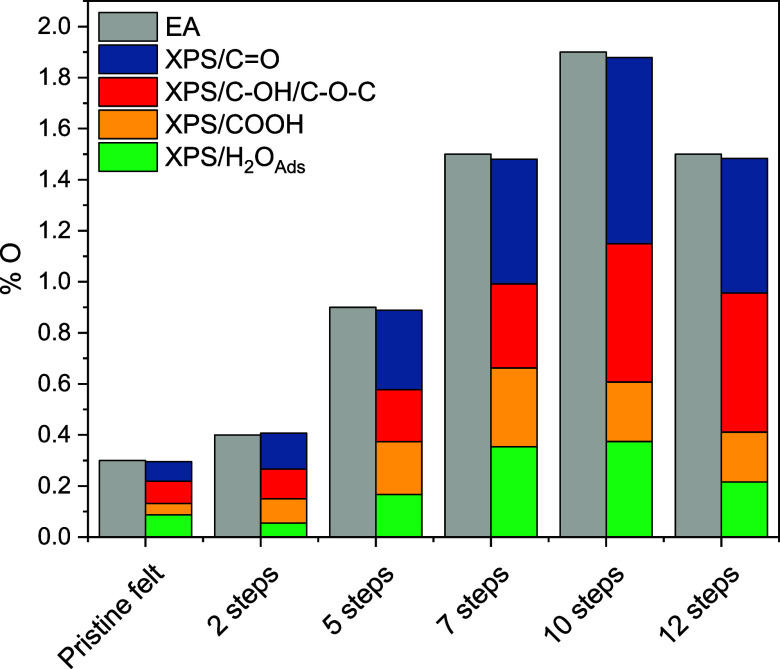
Atomic percentage of oxygen determined by elemental analysis and XPS. Different colored columns represent the oxygen species of each functional group.

Figure 4 shows the total oxygen content and the distribution of species determined by XPS (deconvoluting O 1s spectra) compared with the bulk oxygen content (obtained from EA). The oxygen content increases with an increasing number of impregnation steps, reaching a maximum value of 1.9% at 10 steps. This oxygen increment matches with the amount of rGO deposited (Table 1), indicating that the oxygen content is promoted by the incorporation of graphene sheets into the felt filaments, as has been previously shown in the SEM images (Figure 2). The total amount of surface oxygen is quite similar to the oxygen content in the bulk of the felt, confirming that all the felts present a nearly homogeneous distribution of oxygen, which means that the electrochemically active sites are present over the entire area of the electrode. In addition, the evolution of oxygen content on the surface and in the bulk indicates that the maximum assimilation of rGO by the felt occurs at five impregnation steps, where the differences in the oxygen content are not relevant.

The comparison of the different species distribution shows how the incorporation of C–OH and COOH species is favored between 7 and 10 steps, indicating that this type of oxygen species is incorporated into the carbon felt by the deposition of rGO. In addition, the amount of adsorbed water increases with the number of impregnation steps, indicating higher wettability in those felts with a higher amount of rGO, a parameter that is expected to have a great influence on the performance of FB systems.

In fact, the change in the wettability properties of the pristine felt is clearly evidenced in the following video sequence. When the drop is deposited on the surface of the pristine felt (Figure S5, left), it remains intact on its surface, indicating some degree of hydrophobicity. However, when water drops are deposited on the rGO-modified felt (Figure S5, right), they permeate into the interior of the felt. This shows that the impregnation of the felt with rGO-PEG increases its hydrophilicity, favoring the water/felt interphase contact and the diffusion of the aqueous electrolyte inside the felt. Hydrophilicity can also help to prevent the retention of air bubbles during the assembly of the flow cell and improve electrolyte flow distribution through the electrode.

Carbon felts were further investigated by X-ray diffraction, as shown in Figure S3 in the Supporting Information. The diffractogram shows how the pristine felt presents a main peak at 2Θ = 26.5° corresponding to the (002) plane of graphitic carbon, the majority in the composition of the carbon felt, while no peak can be seen at 11.6° corresponding to the (001) plane of graphene oxide.^52^ Once the felt is impregnated with GO and PEG in the diffractogram, in addition to the peak at 26.5° of graphitic carbon, two peaks at 19.25° and 23.40° corresponding to the (120) and (032) planes of PEG can be seen.^53^ After pyrolysis, the characteristic PEG peaks disappear, indicating that PEG is degraded during the thermal process, resulting in a diffraction profile very similar to that of pristine felt, where graphitic carbon predominates.

**Figure 5:**
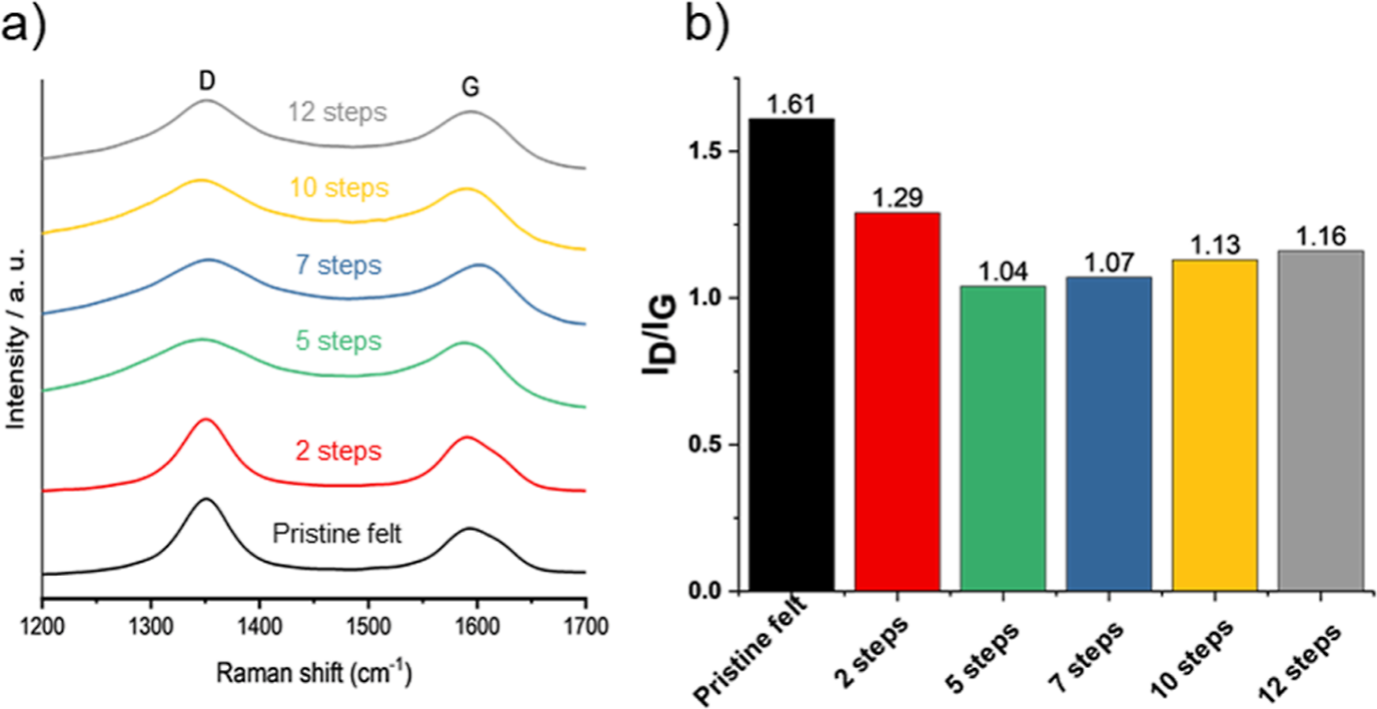
Raman analysis for the different graphite felts modified with rGO/PEG upon successive impregnation steps. (a) Raman spectra and (b) *I*_D_/*I*_G_ ratio.

Carbon felts were also studied by Raman spectroscopy. The Raman spectra shown in Figure 5a display two peaks: one in the range from 1320 to 1360 cm^–1^ (known as the D-band) and another ranging from 1580 to 1600 cm^–1^ (known as the G-band). The G-band is a result of adjacent atoms moving in opposite directions perpendicular to the plane of the graphitic sheet, whereas the D-band (also known as a disordered band) corresponds to atoms moving in radial directions in the plane of the graphitic sheet that appear as a result of dislocations in the lattice, deriving its name from the fact that these are “disorder-induced modes”.

The intensity of the D peak relative to the G peak (*I*_D_/*I*_G_) can, therefore, be used to determine the degree of disorder in the sample,^54^ as shown in Figure 5b. In rGO-modified felts, the ratio of D and G band intensities (*I*_D_/*I*_G_) is lower than that of the pristine felt since the impregnation of rGO on the felt increases the proportion of graphitic carbon coming from the ordered carbon layers of rGO. In particular, this phenomenon is more evident in the five impregnation steps, which shows the lowest *I*_D_/*I*_G_ ratio. Upon successive impregnation steps, the amount of rGO remains stable, as has been reported in Table 1, but the increased content of PEG in the subsequent impregnation could promote the formation of oxygenated species in the rGO sheets and the pristine felt matrix, which could explain how the *I*_D_/*I*_G_ ratio increases with increasing number of deposition steps since a higher amount of oxygen implies a large number of defects (higher intensity of the D band). A greater number of defects in the carbonaceous matrix generated by the oxygenated groups incorporated during the annealing process in the presence of PEG implies a greater number of electrochemically active sites at the electrodes catalyzing the FB reactions.^19,27,34^

### 3.2 Electrochemical Characterization

#### 3.2.1 Vanadium Electrolytes

**Figure 6:**
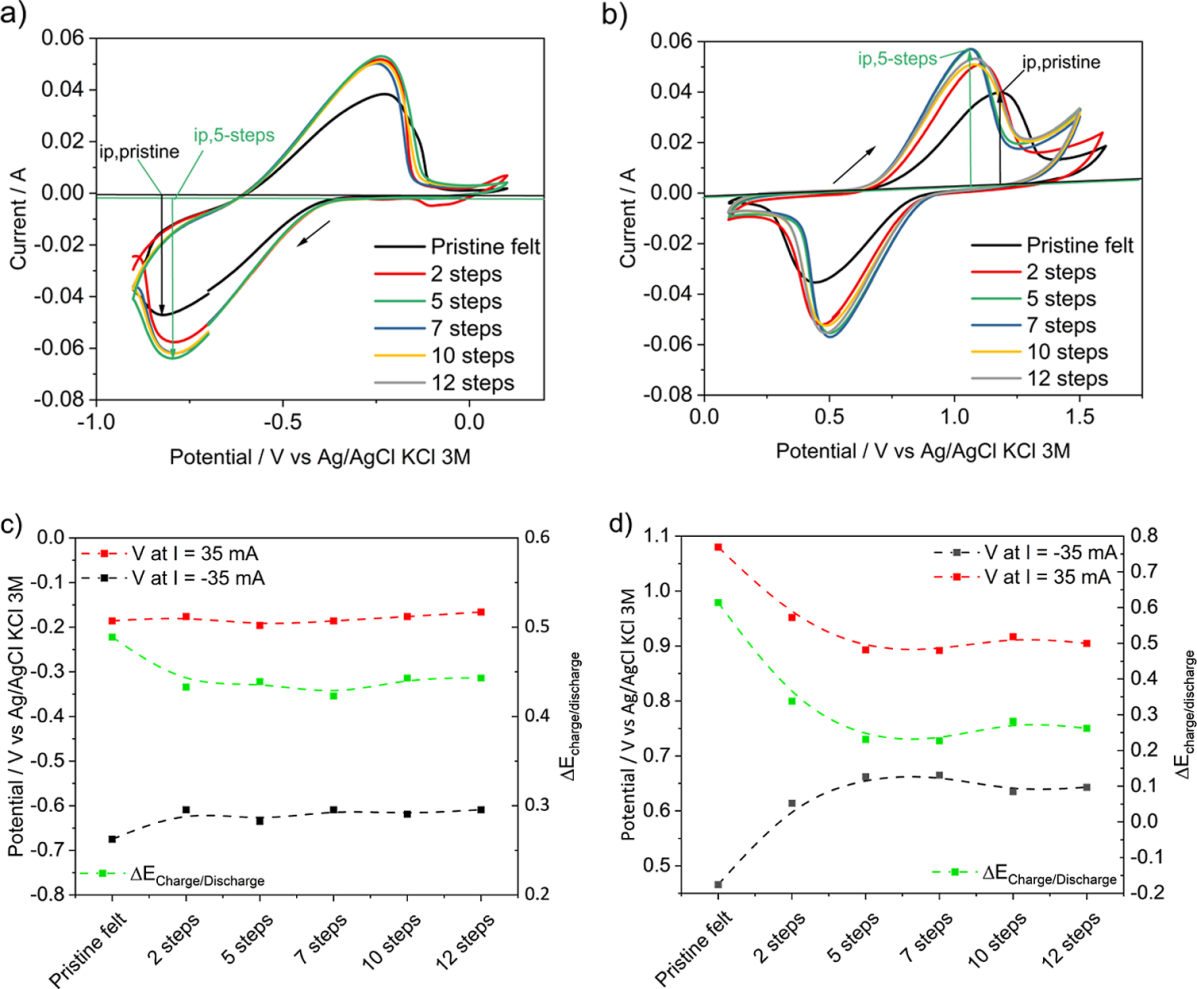
Cyclic voltammetries for the (a) negative (V^2+^/V^3+^) and (b) positive (VO^2+^/VO_2_^+^) electrodes of a VFB (0.05 M VOSO_4_ in 1.0 M H_2_SO_4_, scan rate 5 mV s^–1^); potential at which the current in the voltammetry is 35 mA for the charge and discharge reactions of a VFB for (c) the negative and (d) the positive electrode.

Figure 6 shows the cyclic voltammograms for the modified felts in comparison to that for the pristine felt for the reactions of the negative electrode (Figure 6a, V^2+^/V^3+^) and of the positive electrode (Figure 6b, VO^2+^/VO_2_^+^) of a VFB in a three-electrodeconfiguration. The enhancement is 36.3% for the reduction peak of the V^2+^/V^3+^ couple and 47.7% for the oxidation peak of the VO^2+^/VO_2_^+^ couple. The numerical values of the peak currents are given in Table S2. All of the modified felts afford improved catalytic activity compared to the pristine one, as shown by a higher peak current and closer peak potentials, the latter indicating a higher electrochemical reversibility. The oxidation and reduction of vanadium species display also higher peak currents for the modified electrodes at potentials close to the equilibrium potential of the negative and positive electrodes (*E*^0^ = −0.47 and 0.79 V vs Ag/AgCl, respectively). As the number of impregnation steps increases, the peak potentials become closer to the equilibrium potentials for both reactions. The enhancement of the catalytic activity is more pronounced in the felt with five impregnation steps. The functionalization of the felt with more impregnation steps (7, 10, or 12 steps) does not further increase the activity of the electrode, probably due to a decrease in electrical conductivity as a result of an excess of oxygen functional groups.^34,55,56^ As previously stated, modification of the pristine felts with rGO/PEG creates surface oxygen groups and a rougher surface. Both characteristics have been reported to favor the catalytic performance of the electrodes toward the vanadium redox reactions.^39^

To further assess the catalytic activity of the modified felts for the vanadium reactions, electrode potential values were taken from the voltammograms at fixed current points comparable among the various signals. Figure 6c,d shows the electrode potentials at a current of 35 mA for the charge and discharge reactions for both electrodes (negative and positive, respectively), along with the potential difference for both processes (Δ*E*_charge/discharge_). These results show that, for the same current, the potential difference between the reactions related to charge and discharge processes decreases with the modification of the felt, which would improve the voltage efficiency of a flow battery. This positive effect increases substantially up to a number of five impregnation steps, after which these charge and discharge potentials remain practically unchanged. This observation is consistent with the peak height behavior mentioned above and means that the felt with five impregnation steps has the best composition for this application.

Interestingly, Figure 6c,d clearly shows how the positive effect of incorporating rGO-PEG to the carbon felt is much more pronounced in the positive electrode reactions (VO^2+^/VO_2_^+^) with approximately 350 mV enhancement versus the pristine felt (in terms of Δ*E*) in comparison to the negative electrode (V^2+^/V^3+^), with less than 50 mV improvement with respect to the pristine felt. The electrocatalytic activity is much more pronounced for the positive electrode reactions because, as discussed above, it is known that the limiting step in this type of reaction is the transfer of oxygen atoms.^39^ This oxygen transfer can be favored by the addition of oxygen-rich materials like the ones investigated in this work, while for the negative electrode reactions, the main influence is given by the –C–OH groups, with the rest of the oxygenated groups being of lesser significance.

#### 3.2.2 2,7-AQDS Electrolyte

**Figure 7:**
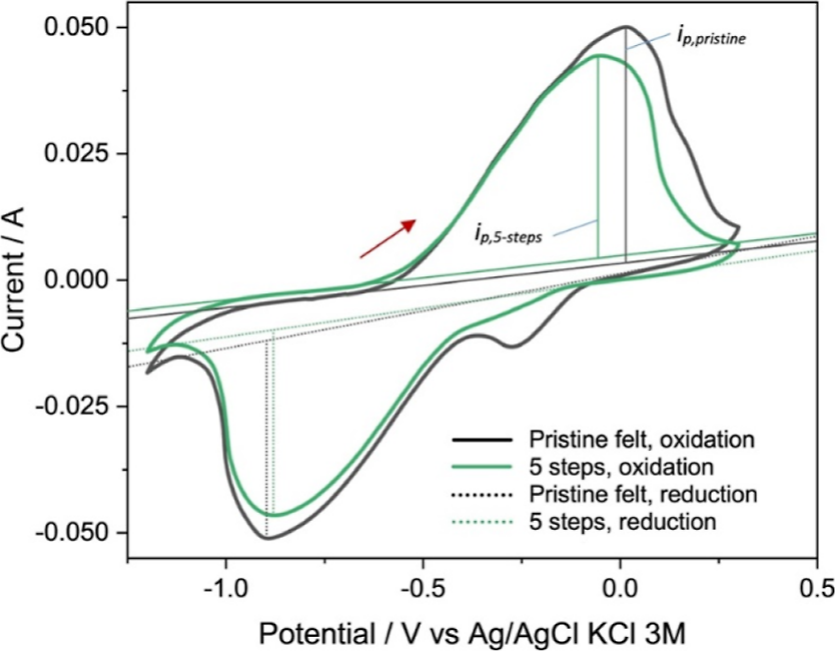
Cyclic voltammetry of the 2,7-AQDS electrolyte with pristine and modified carbon felt (0.05 M 2,7-AQDS in 1.0 M (NH_3_)_2_SO_4_, scan rate 5 mV s^–1^).

The modified felts were also evaluated in a three-electrode cell to study their electrocatalytic activity as an electrode for the redox couple reaction of 2,7-AQDS by using cyclic voltammetry (Figure 7). Having established that five impregnation steps provided better results with the vanadium system, the effect of the rGO-modification on the 2,7-AQDS was tested directly with this felt composition.

As seen in Figure 7, the oxidation peak currents are lower by approximately 14.1% at the modified felt in comparison to that at the pristine felt (Table S3). Moreover, this difference is proportionally less intense than in the case of the VO^2+^/VO_2_^+^ reactions, see Figure 6b, which shows a peak current 47.7% higher at the rGO-modified felt (see also Table S2). The behavior for 2,7-AQDS was confirmed also in sulfuric acid solution, showing a peak current diminution of 8.1% for the oxidation by the modified felt (Figure S6). The fact that the peak current is lower with the modified felt is attributed to the limited diffusion of the 2,7-AQDS species in the diluted electrolyte to the porous electrode. This is supported by the work of Emmel et al.,^47^ who determined the Stokes radius of 2,7-AQDS as 5.6 Å in contrast to 2.8 Å for VOSO_4_ in acidic conditions and stated that transport of the organic molecule is hampered within the porous matrix of a felt electrode.^47^ A bulky molecule such as 2,7-AQDS has has a similar diffusivity to both the pristine and the rGO-modified felts. Moreover, a voltammetry at a felt is controlled by a finite diffusion among and around the fibers,^57^ unlike the ideal planar disk electrode. Therefore, the activity of the electrode surface may not be the determining step of the reaction when diffusion is the slower step in such nonideal voltammograms.^12^ These effects do not significantly influence the operation of a flow cell with continuous convection, as seen by many examples of OFBs using felt electrodes. Still, a strategy to improve the diffusivity of 2,7-AQDS in flow cells could combine a rise in electrolyte velocity, an improvement in the mass transport coefficient of the electrode or cell design, and possibly a moderately high temperature.

The voltammograms in Figure 7 also indicate that the peak currents for the reduction of 2,7-AQDS are higher than those for its oxidation process (Table S3.). This is consistent with the fact that reduction rates of 2,7-AQDS are slower, close to neutral pH, in contrast to alkaline pH.^46^ Also, the peak current for the reduction is 7.6% lower for the modified carbon felt compared to that for the pristine felt, in accordance with the above discussion on the Stokes radius of 2,7-AQDS. As a side note, the voltammogram of pristine felt in the 1.0 M (NH_3_)_2_SO_4_ electrolyte showed a small peak near −0.25 V vs Ag/AgCl (Figure 7). This was not observed in any of the experiments with vanadium redox couples or when 2,7-AQDS was added to the supporting electrolyte. Despite purging the electrolyte with nitrogen, the potential is close to oxygen reduction on glassy carbon modified with carbon nanotubes,^58^ pointing out the possibility of traces of absorbed oxygen reacting over the modified catalytic electrodes as well as the need for more investigation on the behavior of carbon felts in solutions containing ammonium ions.

#### 3.2.3 Evaluation in a Vanadium Flow Battery

**Figure 8:**
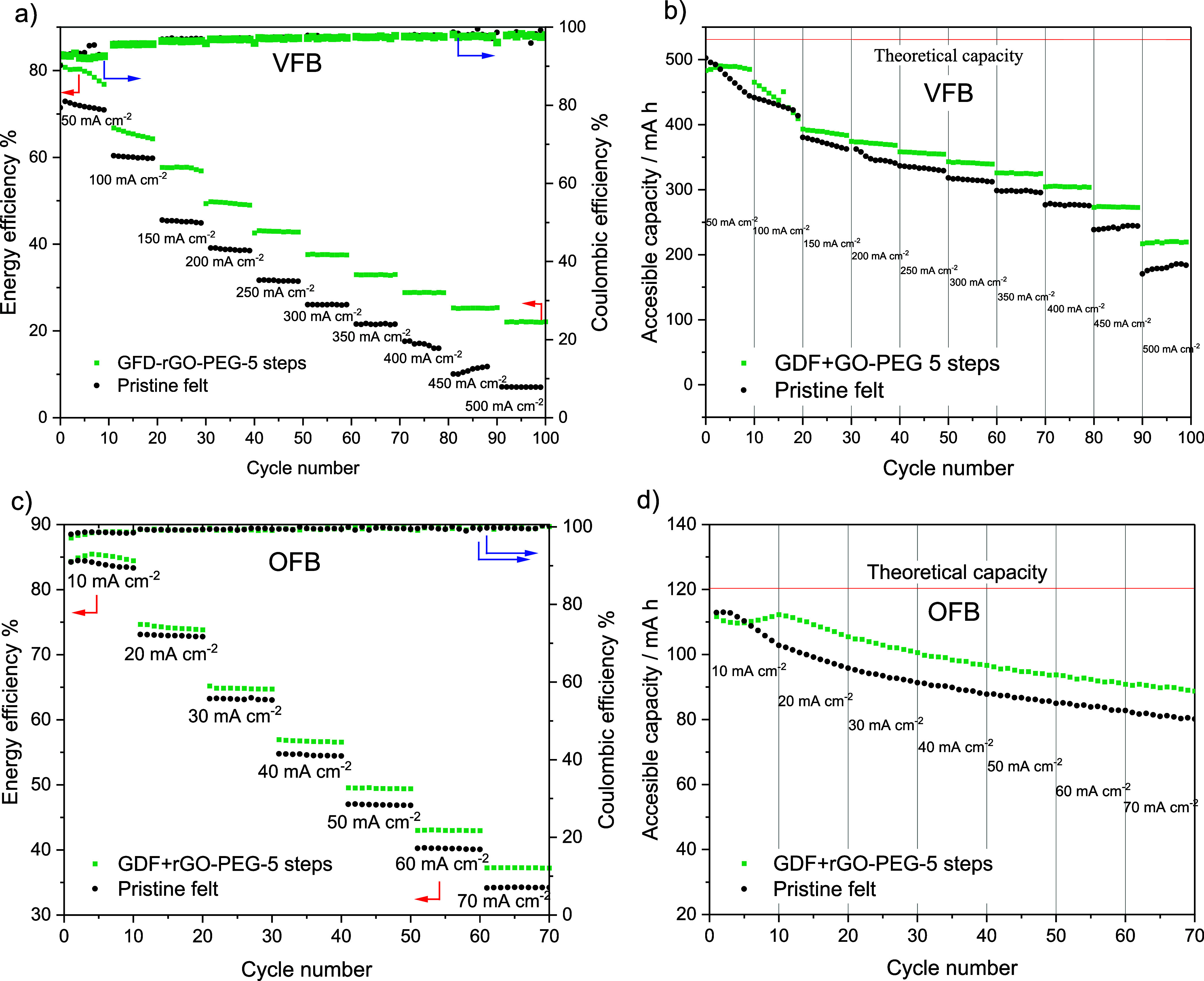
Test on a vanadium flow battery with both redox couples at a concentration of 0.4 M in 2.0 M H_2_SO_4_ and 0.05 M H_3_PO_4_. Effect of current density (10 cycles per value) on (a) energy efficiency and Coulombic efficiency and (b) accessible capacity. Duration of experiment: 52 h. Test on an organic flow battery using a solution of 2,7-AQDS 0.2 and 1.0 M (NH_3_)_2_SO_4_ as the negative electrolyte and Na_4_[Fe(CN)_6_] 0.2 and 1.0 M (NH_3_)_2_SO_4_ as the positive electrolyte. Effect of current density (10 cycles per value) on (c) energy efficiency and Coulombic efficiency and (d) accessible capacity. Duration of experiment: 35 h.

The felt with five impregnation steps was chosen for the flow cell studies in a single-cell battery configuration since it provided the best catalytic activity in the three-electrode cell experiments. Figure 8a shows both the energy and Coulombic efficiency of a VFB cell, along with the discharge capacity upon cycling. Ten charge–discharge cycles at different current densities were performed to evaluate the behavior of the electrodes under increasing current density, showing the typical overall decay in efficiency resulting from the ohmic components in the cell along the increased activation and mass transfer overpotentials. Cell voltage vs time profiles are shown in Figure S8 and show the typical decrease in cycle duration with increasing current density. The results in this flow cell are validated by the fact that the energy efficiency of ca. 60% at 100 mA cm^–2^ for the pristine felt is the same as that reported for a laboratory VFB (Nafion 115 membrane) with a similar 5 mm-thick, nonmodified felt.^59^ However, as shown in Figure 8a, the felt modified with rGO/PEG (GFD-rGO-PEG-5steps) affords a higher activity than the pristine electrode in the flow cell, resulting in enhanced energy efficiency at all of the evaluated current densities. This improvement in the average energy efficiency goes from 12.4% at 50 mA cm^–2^ to 26.8% at 200 mA cm^–2^. Proportionally speaking, the enhancement is more intense at higher current densities, as driven by mass transfer limitations, but the energy efficiencies fall below 40%, which is well into impractical values. Coulombic efficiency did not change significantly between pristine and modified felt, with both their values rising from just over 95% to 98% over the rising range of current density; again, these are common values in comparable laboratory VFBs due to crossover and atmospheric oxygen,^59^ which explain the capacity loss slopes observed in the experiments of Figure 8b. The improvement in the energy efficiency performance of the rGO-modified felt compared to that of the pristine felt is caused by a higher presence of oxygenated groups on the surface of the electrodes that catalyze the redox reactions of both the V^2+^/V^3+^ and VO^2+^/VO_2_^+^ redox couples,^19^ which combined with a higher surface area and hydrophilicity of the modified felt compared to that of the pristine commercial felt. As shown by Sun and Skyllas–Kazacos,^60,61^ the enhanced wettability and electrochemical activity of the treated graphite felt are a result of a rise in the number of C–OH and C=O functional groups on its surface.

As shown in Figure 8b, the higher efficiency of the modified felt also translates into an improvement in the accessible capacity of the battery for a given current as the cell was operated in galvanostatic mode and hence lower overpotentials at more active electrodes delay reaching the cell voltage cutoff conditions. Average accessible capacities rose by 5.4% at 200 mA cm^–2^ to 8.9% at 400 mA cm^–2^. It should be noted that the accessible capacity displayed clear transient effects at the two lowest evaluated current densities, likely due to the progressive oxidation and reduction of the additional surface functional groups supplied by the rGO in combination with membrane stabilization under the imposed charge–discharge conditions.

To recap, the higher oxygen content of the modified felt, along with its rougher surface and hydrophilicity, which favor the interaction of vanadium ions with the surface of the felt, is responsible for the enhanced performance of the FB with the modified felt electrodes. This indicates that the rGO-modified felt allows operating the battery at high current densities while maintaining high energy efficiency, achieving improvements equal to or superior to similar works reported in the recent literature using similar flow systems and the same commercial felt as pristine felt.^30,62^ The modifications made to the pristine felt in this work can be extrapolated to any of the other carbon felts of the same chemical composition.

#### 3.2.4 Evaluation in an Organic Flow Battery

Having established the functionality of the rGO-modified electrodes on the vanadium system as a benchmark, the attention was then turned to the anthraquinone/ferrocyanide flow battery, considering the felt modified by five impregnation steps. The energy efficiencies for this system using pristine and rGO-modified felt are compared in Figure 8c. Cell voltage vs time profiles are shown in Figure S9. As with the case of the inorganic FB, the flow cell using the modified felt affords higher values than when using pristine felt, and the relative improvement increases as the charge and discharge current density is augmented. The enhancement in the average energy efficiency rises from approximately 3.2% at 10 mA cm^–2^ to 16.1% at 50 mA cm^–2^. The performance improvement in the anthraquinone/ferrocyanide FB with the rGO-modified felt is due to a combination of the presence of catalytic oxygenated groups at its surface, enhanced wettability, and probably extended surface area, as in the case of other modified electrodes.^19^ The improvement is modest compared to that of the VFB case because transport of 2,7-AQDS toward the porous felt is limited by its relatively large radius,^47^ as discussed in the cyclic voltammetry results. Yet, contrary to the nonideal voltammetry at felts in the quiescent solutions, the activity of the GO modification reveals itself as efficiency and accessible capacity enhancement in the flow battery since the continuous mass transport supplied by the flowing electrolyte eliminates the diffusion restrictions imposed by the fiber electrode.

These efficiency values can be contrasted to other anthraquinone-based semiorganic FBs. For instance, the AQDS/ferrocyanide in an ammonium ion electrolyte reported by Fenini et al. displayed essentially the same energy efficiency of 60% at 100 mA cm^–2^ for unmodified felt with a thinner Fumasep 630 membrane,^46^ while an alkaline system based on anthraquinone with 2–2-propionate ether anthraquinone (2–2PEAQ)/ferrocyanide by Amini et al. showed values of 62% at 50 mA cm^–2^.^63^ Regarding the Coulombic efficiencies, these are indistinguishable between pristine and modified felt, being in all cases over 98% and approaching 100%. These values reflect the capacity retention found in most laboratory OFBs.^10^ The presence of rGO at the felt electrode allowed us also to extend the capacity utilization of the flow cell, as seen in Figure 8d. The accessible capacity showed average enhancement values of 12.2% at 20 mA cm^–2^, reaching 12.9% at 50 mA cm^–2^. Again, this is explained by the reduction of overpotentials in the galvanostatic charge–discharge regime by the presence of the active rGO and perhaps the diminution of the local current density at the fibers due to their larger surface area.

Figure 8d also shows that the accessible capacity is continuously lost during the experiments with 2,7-AQDS. This is mainly a result of atmospheric oxygen diffusion into the electrolyte circuit despite the continuous supply of 5.0 nitrogen and the liquid-hermetic equipment, which was nevertheless operated outside a glovebox. The reader is referred to the discussion on the adequacy of electrolyte circuits for OFBs offered by Thurston et al.^10^ More specifically, the capacity of the limiting 2,7-AQDS negative electrolyte is lost through the reaction of its reduced form with oxygen, which produces the oxidized form of the organic molecule and an oxygen radical anion.^64^ It is certainly desirable to perform future similar experiments in a fully inert environment alongside larger volumes of electrolytes.

The enhancement of carbon felt by the rGO modification can be compared to a single example of “etched” felts applied to the DHAQ/ferrocyanide system.^41^ In that work, PAN and Rayon-based felts were thermally treated after soaking them in NiCl_2_, FeCl_3_, or CoCl_2_ aqueous solutions. However, only pristine and nickel-etched felts were tested in a flow cell at an unknown current density. The nickel-etched PAN-based felt increased the Coulombic efficiency by 5% in comparison to the pristine felt in the DHAQ/ferrocyanide battery. It must be mentioned that the Coulombic efficiency recorded during each of the 40 cycles was ca. 70%, indicating a severe loss of capacity, while the energy efficiency was not reported. In contrast, the rGO-modified PAN-based carbon felt used in this work afforded an energy efficiency enhancement of 16.1% at 50 mA cm^–2^, at which the Coulombic efficiency was >98%. As discussed above, the energy efficiency improvement is mainly due to rGO, while the higher Coulombic efficiency is due to a better capacity retention. A strict comparison between these two works is not possible, but the rGO-modification seems more adequate at face value. The evaluation of electrocatalyst-modified electrodes in OFBs should aim toward agreed evaluation conditions and figures of merit.

The observed effect of rGO-modified electrodes in the overall flow cell performance shows promise to tackle one of the main challenges in OFBs, that is, the limited operational current (and power) density compared to vanadium systems resulting from high area-specific resistance, which is a consequence, in most cases, of poorly conductive aqueous electrolytes and resistive ionic exchange membranes.^65^ Combined with relatively high reactant costs and capacity retention, low currents represent an important techno-economic challenge for OFBs.^66,67^ Indeed, the energy efficiencies of VFBs can routinely achieve values close to 80% for rectangular-channel flow cells operating at 200 mA cm^–2^.^68^ Having demonstrated the concept of rGO-modified electrodes for OFB, more work should be dedicated to the long-term stability of these electrocatalysts and to the consequences of their presence on the lifetime of the redox active species in combination with flow cells of lower area specific resistance. Stability studies can be fulfilled using symmetric flow cell experiments under combined galvanostatic/potentiostatic control in inert gas gloveboxes,^10^ cell-in-series experiments,^69^ and electrochemical impedance spectroscopy (EIS) coupled to X-ray photoelectron spectroscopy (XPS).^17^

## 4 Conclusions

In summary, commercial carbon felts were modified with reduced rGO and PEG to obtain electrodes with oxygenated functional groups. The amount of rGO was adjusted by performing several GO/PEG impregnation steps, aiming to achieve the highest performance in a VFB benchmark, followed by the first evaluation of the improvement of an OFB based on 2,7-AQDS as a result of catalyst-modified carbon felts. It was determined that surface oxygen groups (five impregnation steps) provided hydrophilicity and a larger surface area to a modified carbon felt, enhancing its catalytic activity toward both the VFB and the OFB in half-cell and full-cell configurations. In the VFB, the effect of the modified felt was more pronounced at the positive electrode since the limiting step in this type of reaction is the transfer of oxygen atoms, which is favored by the addition of oxygen-rich materials. The modification of the carbon felt with five impregnation steps of rGO/PEG provided an enhancement of 26.8% in the energy efficiency of the VFB at 200 mA cm^–2^. Notably, the same rGO-modified felts tested as the electrodes of a 2,7-AQDS-based OFB produced promising results by boosting the energy efficiency in comparison to that of the pristine felt, with an enhancement in energy efficiency of 16.1% at 50 mA cm^–2^. In contrast to the vanadium species, 2,7-AQDS voltammograms in a three-electrode cell showed slightly decreased peak heights with the modified felt as a result of the low diffusivity of the molecule in combination with the finite diffusion among the fibers of the nonideal porous electrode. Together with other improvement strategies, carbon felts modified with nonmetallic electrocatalysts could help OFBs to approach technical feasibility by allowing operation at higher current densities, provided that cost-effectiveness, molecular stability, and capacity retention are maintained. Further work should focus on the durability of the rGO-modified felts in OFBs.

## Supporting Information Available

XPS analysis (high-resolution XPS spectra of the C 1s orbital, high-resolution XPS spectra of O 1s, and percentage of the different carbon and oxygen species); X-ray diffraction and scanning electron microscopy (diffractogram, SEM images, and Video capture); peak current analysis (selected peak currents of Figure 7 and Table S3 and selected peak currents of Figure 8); cyclic voltammetry in acid solution (cyclic voltammetry of the 2,7-AQDS electrolyte); Randles-Sevčik analysis (relation between oxidation/reduction peaks and the square root of the scan rate); and cell voltage vs time plots (effect of current density on the cell voltage of the VRFB and ORFB) (PDF)
Capture of water drops falling on the surface of both the pristine felt (left) and a rGO/PEG-modified felt by 5 impregnation steps (right) (AVI)

## Supplementary Material

ae3c03223_si_001.pdf

ae3c03223_si_002.avi
