# Supplementary material for: Comparison of the Influence of Oxygen Groups Introduced by Graphene Oxide on the Activity of Carbon Felt in Vanadium and Anthraquinone Flow Batteries
Source: ACS Appl Energy Mater. 2024 Mar 18;7(7):2779–90. doi: 10.1021/acsaem.3c03223 (PMC11005476; doi:10.1021/acsaem.3c03223)
Supplement: Supplementary file 1 — ae3c03223_si_001.pdf [file ae3c03223_si_001.pdf]

SUPPORTING INFORMATION

Comparison of the influence of oxygen groups introduced by graphene oxide on the activity of carbon felt in vanadium and anthraquinone flow batteries

Antonio J. Molina-Serrano[1], José M. Luque-Centeno[1], David Sebastián[1], Luis F. Arenas[2,3,*,†], Thomas Turek[2,3], Irene Vela[1], Francisco Carrasco-Marín[4], María J. Lázaro[1,*], Cinthia Alegre[1,*]

[1] Instituto de Carboquímica, Consejo Superior de Investigaciones Científicas-CSIC. C/ Miguel Luesma Castán, 4, 50018, Zaragoza, Spain

[2] Institute of Chemical and Electrochemical Process Engineering, Clausthal University of Technology, Leibnizstraße 17, 38678 Clausthal-Zellerfeld, Germany

[3] Research Center for Energy Storage Technologies, Clausthal University of Technology. Am Stollen 19 A, 38640, Goslar, Germany

[4] Facultad de Ciencias, Universidad de Granada. Avd. de Fuente Nueva, s/n, 18071, Granada, Spain

*Corresponding authors: C.A. (cinthia@icb.csic.es); L.F.A. (arenas@tu-clausthal.de); M.J.L. (mlazaro@icb.csic.es)

[†] Present address: Research Group Applied Electrochemistry & Catalysis (ELCAT), University of Antwerp, Universiteitsplein 1, 2610 Wilrijk, Belgium.

1. XPS analysis

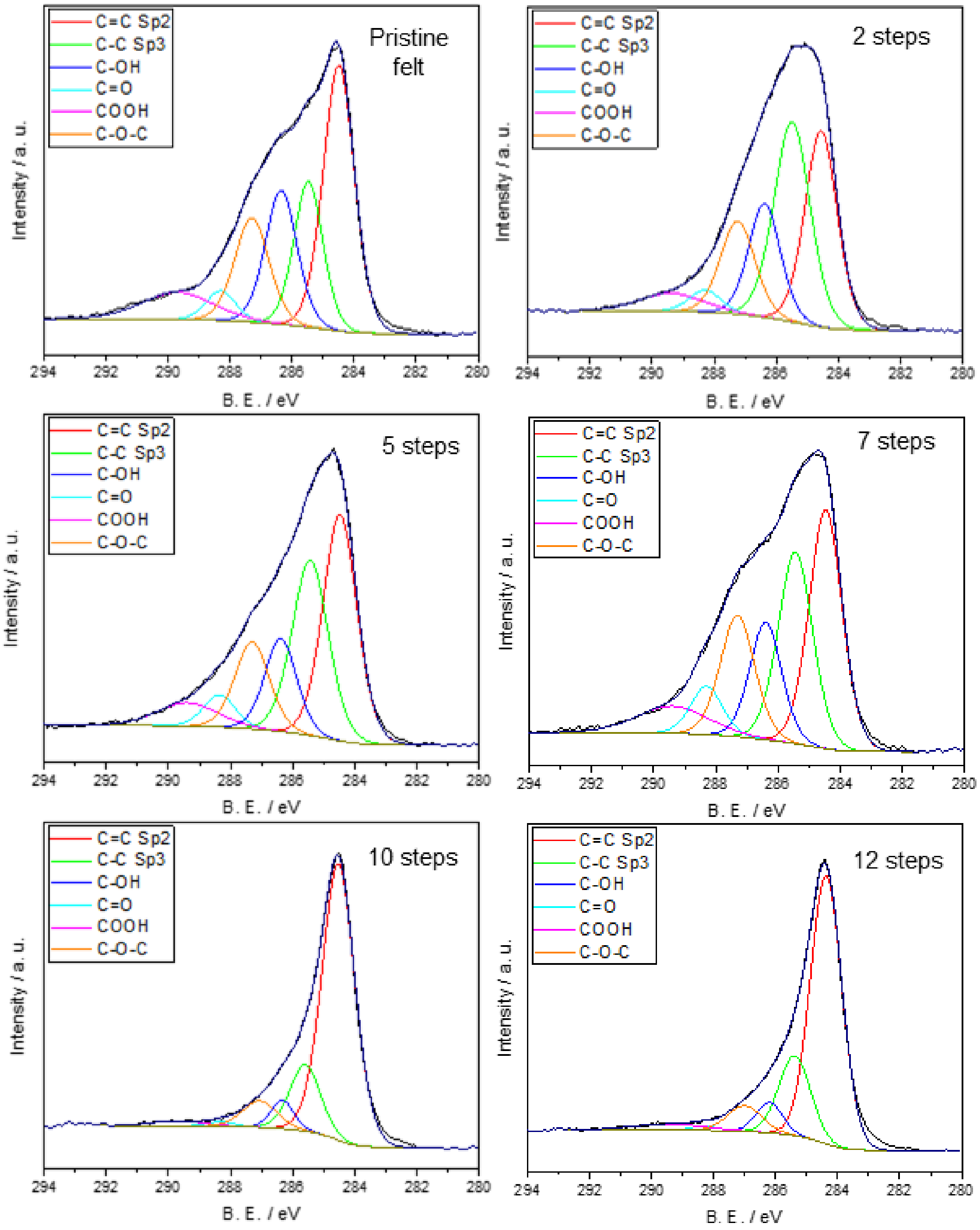

Figure S1. High resolution XPS spectra of C1s for the pristine carbon felt and for the felt with different impregnation steps: 2, 5, 7, 10 and 12.

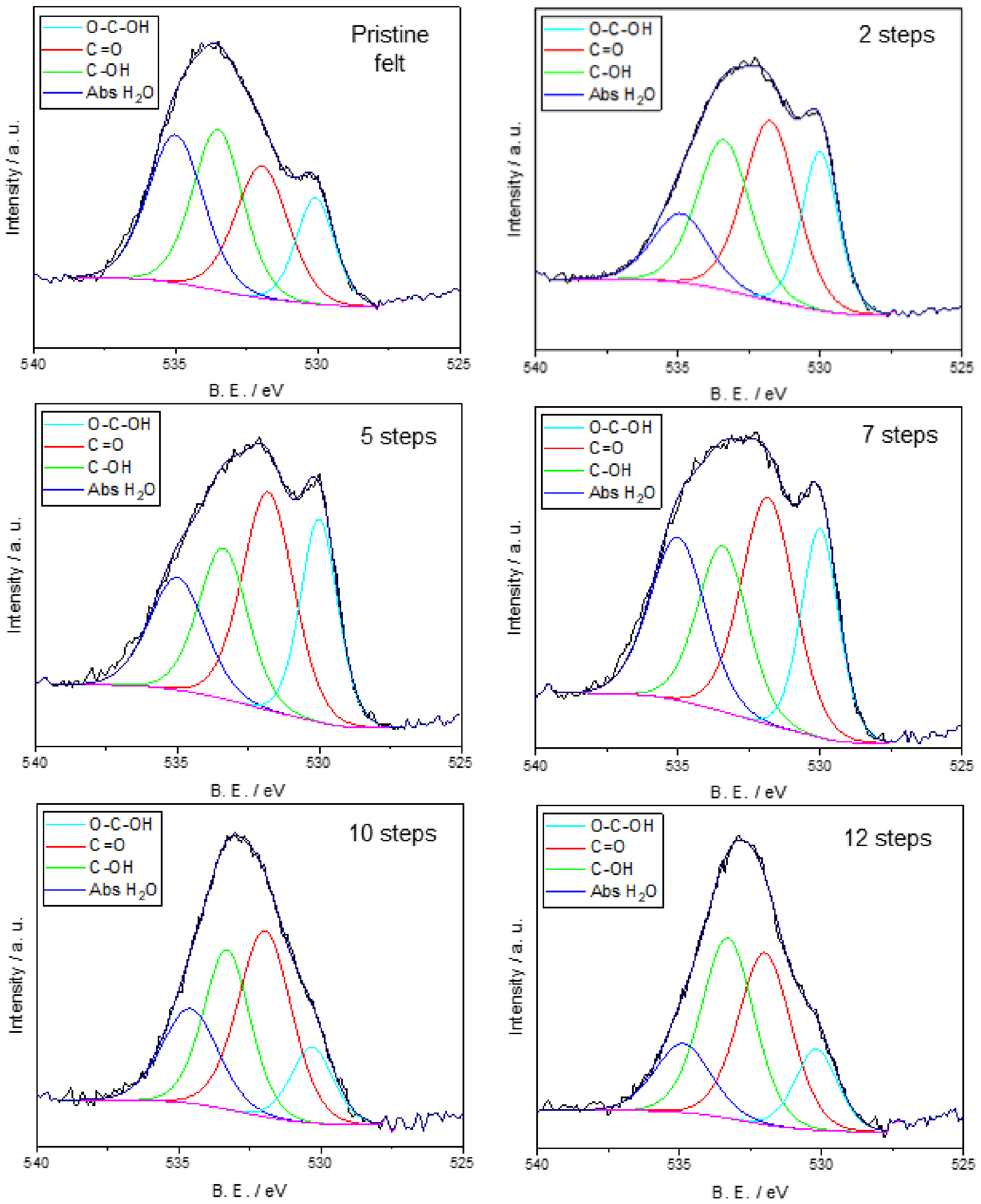

Figure S2. High resolution XPS spectra of O1s for the pristine carbon felt and for the carbon felt with different impregnation steps: 2, 5, 7, 10 and 12.

Table S1. Percentage of the different carbon and oxygen species determined by XPS for the felts (from C1s and O1s deconvolution).

Carbon felt	C species (%)						O species (%)			
	C=C sp^2	C-C sp^3	C-OH	C=O	COOH	C-O-C	O-C-OH	O=C	OH-C/ -O-C	H_2O Ads
**Pristine felt**	36.0	18.1	18.3	3.8	8.4	15.4	14.9	25.6	29.6	29.9
**2 steps**	28.6	32.4	16.2	3.2	5.2	14.4	21.5	35.2	29.3	14.0
**5 steps**	34.3	28.3	13.2	4.6	6.3	13.3	23.4	34.6	22.9	19.1
**7 steps**	30.5	25.5	14.4	6.0	7.5	16.1	21.0	32.5	22.2	24.3
**10 steps**	69.4	15.6	5.0	1.0	2.0	7.0	12.5	38.4	28.8	20.3
**12 steps**	64.2	19.1	6.0	0.9	3.2	6.6	13.3	35.2	36.7	14.8

2. X-ray diffraction and scanning electron microscopy

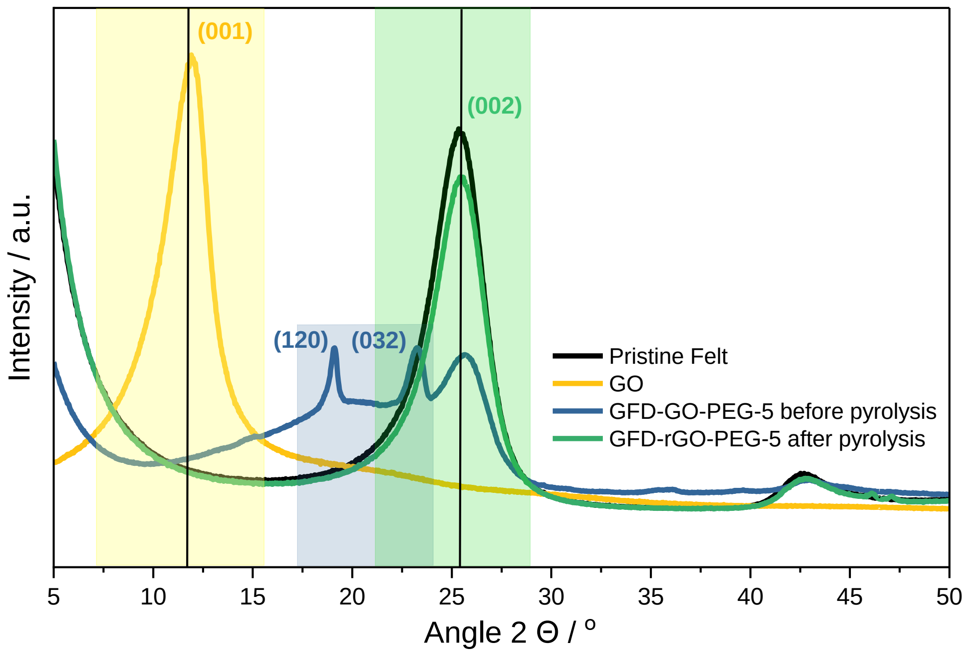

Figure S3. Diffractogram at different stages of synthesis process.

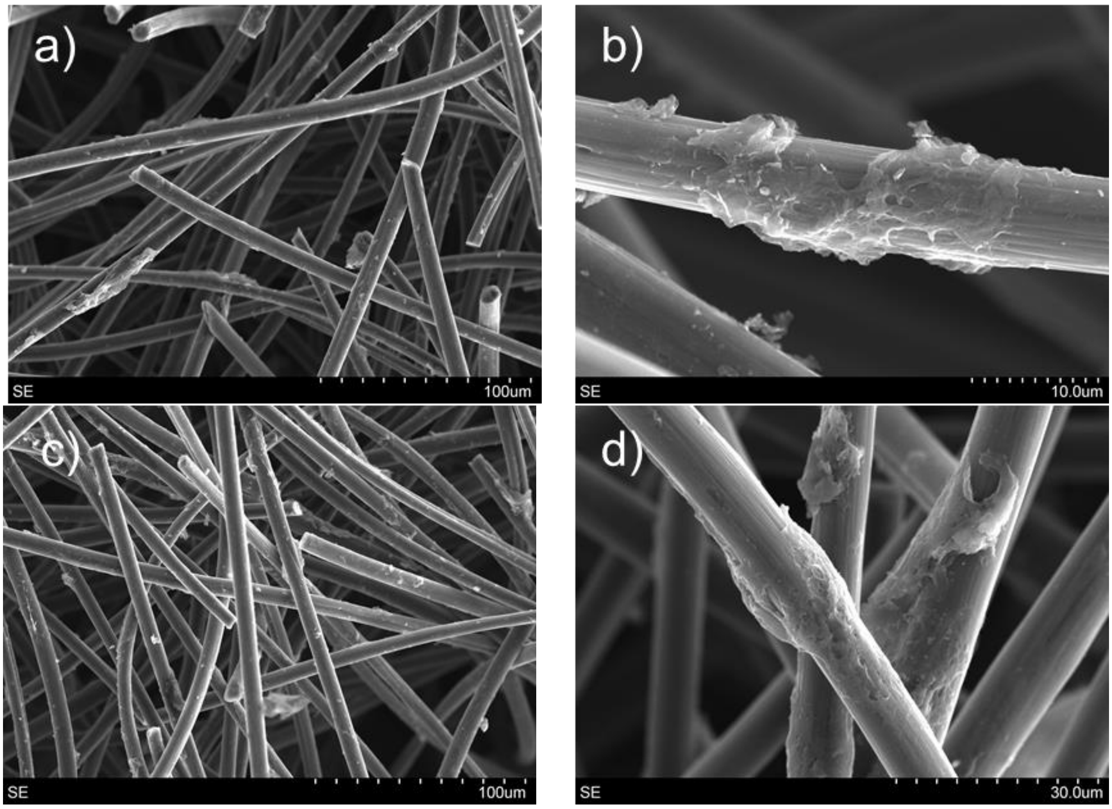

Figure S4. SEM images of rGO-PEG modified graphite felts with five impregnation steps after being used as negative electrode a) and b) and positive electrode c) and d) in a VRFB.

The change in the wettability properties of the pristine felt is evidenced in the following video sequence, where some drops of water were poured on top of the felt (both pristine and rGO-PEG modified felt). When the drop is deposited on the surface of the pristine felt (**Figure S5**, left) it remains intact on its surface indicating some degree of hydrophobicity. Whereas, when water drops are deposited on the rGO-modified felt (**Figure S5**, right), they permeate into the interior of the felt. This shows that the impregnation of the felt with rGO-PEG increases its hydrophilicity, favoring the water/felt interphase contact and the diffusion of the aqueous electrolyte inside the felt. Hydrophilicity can also help to prevent the retention of air bubbles during the assembly of the flow cell and improve electrolyte flow distribution through the electrode.

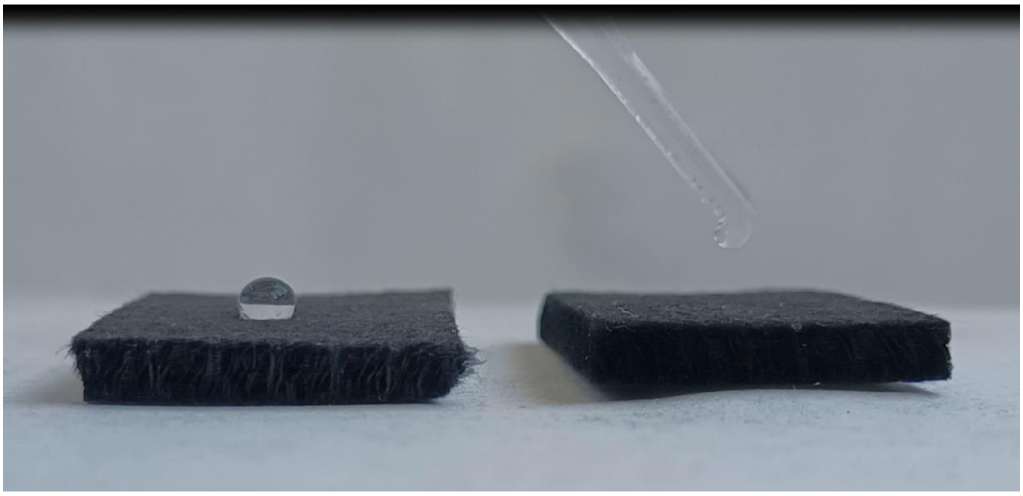

Figure S5. Capture of water drops falling on the surface of both the pristine felt (left) and a rGO/PEG-modified felt by 5 impregnation steps (right).

3. Peak Current Analysis

Table S2. Selected peak currents in the voltammograms of Figure 7: cyclic voltammetries for the a) negative (V^{2+}/V^{3+}) and b) positive (VO^{2+}/VO_2^{+}) electrode of a VFB (0.05 M $VOSO_4$ in 1.0 M H_2SO_4, scan rate 5 mV s^{-1}).

Carbon felt electrode	Peak current, mA	Percentage change vs. pristine felt, %
**a) Pristine felt, reduction ($i_{p,pristine}$)**	46.8	NA
**a) 5 steps, reduction ($i_{p,5\text{-}steps}$)**	63.8	+36.3
**b) Pristine felt, oxidation ($i_{p,pristine}$)**	37.3	NA
**b) 5 steps, oxidation ($i_{p,5\text{-}steps}$)**	55.1	+47.7

Table S3. Selected peak currents in the voltammograms of Figure 8: cyclic voltammetry of the 2,7-AQDS electrolyte with pristine and modified carbon felt (0.05 M 2,7-AQDS in 1.0 M $(NH_3)_2SO_4$, scan rate 5 mV s^{-1}).

Carbon felt electrode	Peak current, mA	Percentage change vs. pristine felt, %
**Pristine felt, oxidation ($i_{p,pristine}$)**	46.7	NA
**5 steps, oxidation ($i_{p,5\text{-}steps}$)**	40.1	-14.1
**Pristine felt, reduction ($i_{p,pristine}$)**	39.5	NA
**5 steps, reduction ($i_{p,5\text{-}steps}$)**	36.5	-7.6

4. Cyclic Voltammetry in Acid Solution

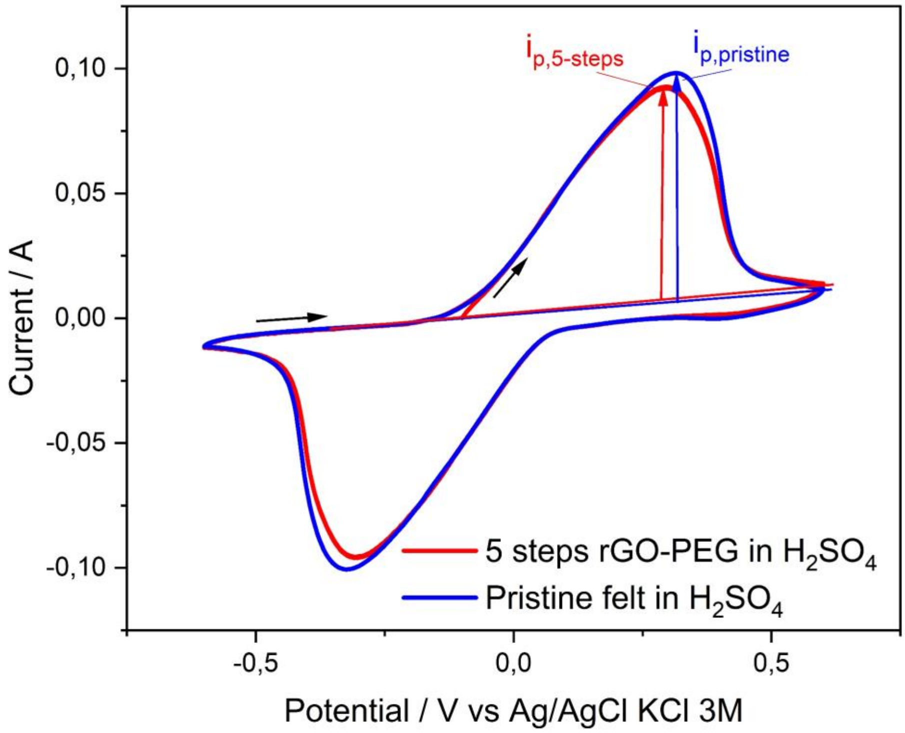

Figure S6: Cyclic voltammetry of the 2,7-AQDS electrolyte with pristine and modified carbon felt (0.05 M 2,7-AQDS in 1.0 M H_2SO_4, scan rate 5 mV s^{-1}). Modified felt was a different batch. For the oxidation peak: $I_{p,pristine}$ is 91.8 mA and $I_{p,5\text{-steps}}$ is 84.3 mA, the latter value being 8.1 % lower than the first.

5. Randles-Sevčik Analysis

To estimate the comparative surface area of the pristine and modified felts, a Randles-Sevčik was performed on the voltammograms with the knowledge of the reported diffusion coefficient. For an irreversible system the relevant equation is:

$$I_p = 0.4463nFAC\left(\frac{nFvD}{RT}\right)^{1/2} \qquad \textbf{Equation (1)}$$

The Randles-Sevčik equation, at 25ºC and summarizing the constants, can be expressed as follows:

$$I_p = 2.99\times10^5 n(n\alpha)^{1/2} AC_0 D_0^{1/2} v^{1/2} \qquad \textbf{Equation (2)}$$

Where:
I_p - peak current (A)
n - number of electrons per molecule oxidized or reduced
α - transfer coefficient
n_a - number of electrons involved in rate-determining step
A - electrode area (cm^2)
C_0 - solution concentration ($mol\cdot cm^{-3}$)
D_0 - diffusion coefficient ($cm^2\cdot s^{-1}$)
v - scan rate ($V\cdot s^{-1}$)
F - Faraday constant ($s\cdot A\cdot mol^{-1}$)
R - gas constant ($V\cdot A\cdot s\cdot K^{-1}\cdot mol^{-1}$)
T - temperature (K)

For the reaction:
$VO^{2+} + H_2O \leftrightarrow VO_2^+ + 2H^+ + e^-$
n=1, αn_a=0.5, (generally considered in literature)
A plot of i_p vs. $v^{1/2}$ should give a straight line with slope proportional to D_0.
Slope must be equal to:
$2.99 \times 10^5 (0.5)^{0.5} AC_0 D_0^{0.5}$,
Where $C_0 = 5.0 \times 10^{-5}$ mol cm^{-3}, and $D_0 = 3.2 \times 10^{-6}$ cm^2 s^{-1} [1].

We can calculate the active surface area of the felt used as working electrode:

The results for the oxidation peaks are **56.64 cm^2** and **60.93 cm^2** for the pristine felt and GFD+rGO-PEG 5 steps respectively. If the same calculation is repeated with the reduction peaks the values are **66.19 cm^2** for the pristine felt and **35.80 cm^2** for GFD+rGO-PEG 5 steps.

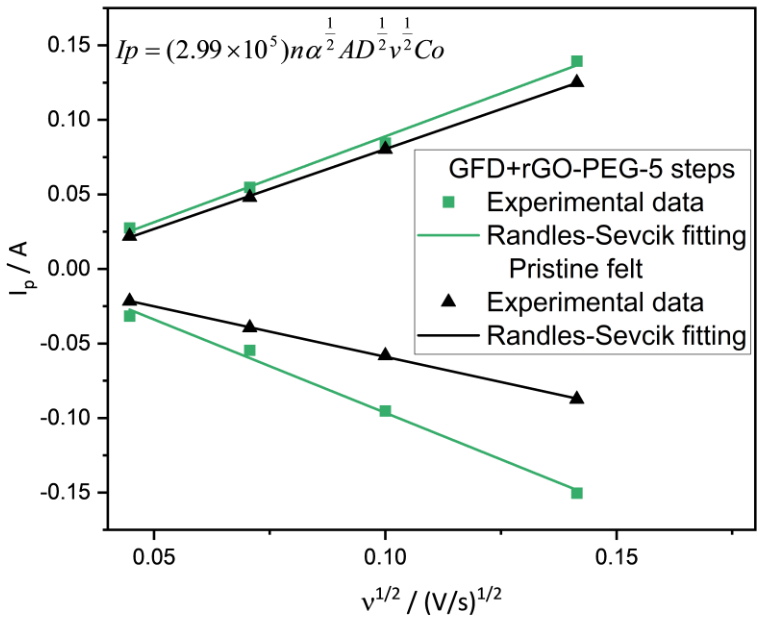

Figure S7. Relation between the oxidation and reduction peaks current and the square root of the scan rate from 2 to 200 mV s^{-1}.

6. Cell Voltage vs. Time Plots

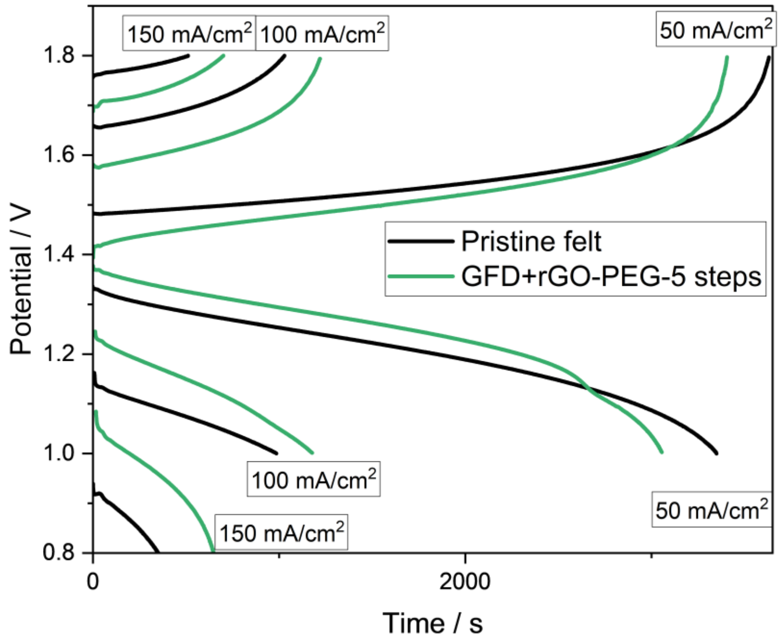

Figure S8. Effect of current density on the cell voltage vs. time profiles of the VRFB. Current density values: 50, 100 and 150 mA/cm^2.

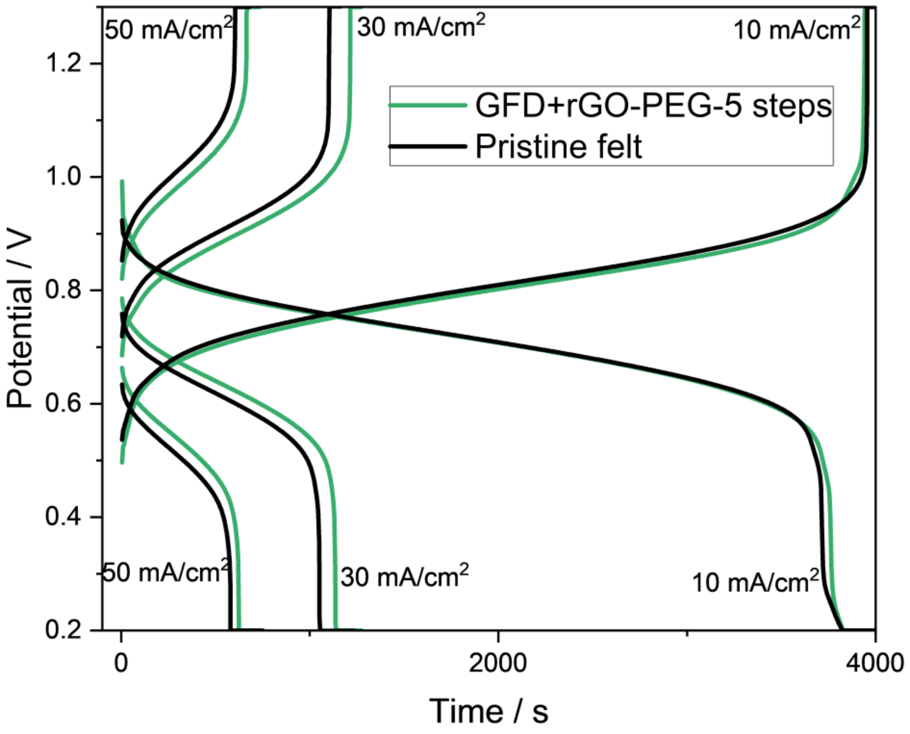

Figure S9. Effect of current density on the on the cell voltage vs. time profiles of the 2,7-AQDS organic flow battery. Current density values: 50, 100 and 150 mA/cm^2.

References

(1) Barranco, J. E.; Cherkaoui, A.; Montiel, M.; González-Espinosa, A.; Lozano, A.; Barreras, F. Analysis of the Electrochemical Performance of Carbon Felt Electrodes for Vanadium Redox Flow Batteries. *Electrochim. Acta* **2023**, *470* (December 2022). https://doi.org/10.1016/j.electacta.2023.143281.
